# Myotubularin-Related Phosphatase 3 Promotes Growth of Colorectal Cancer Cells

**DOI:** 10.1155/2014/703804

**Published:** 2014-08-19

**Authors:** Bo'an Zheng, Xiaojun Yu, Rui Chai

**Affiliations:** ^1^Department of Colorectal Surgery, Zhejiang Provincial People's Hospital, Hangzhou, Zhejiang 310014, China; ^2^Department of Gastroenterological Surgery, Zhejiang Provincial People's Hospital, 158 Shangtang Road, Hangzhou, Zhejiang 310014, China

## Abstract

Due to changes in lifestyle, particularly changes in dietary habits, colorectal cancer (CRC) increased in recent years despite advances in treatment. Nearly one million new cases diagnosed worldwide and half a million deaths make CRC a leading cause of cancer mortality. In the present study, we aimed to investigate the role of myotubularin-related phosphatase 3 (MTMR3) in CRC cell growth via lentivirus-mediated small interfering RNA (siRNA) transduction in human colon cancer cell lines HCT116 and SW1116. The effect of MTMR3 knockdown on cell growth was evaluated by MTT, colony formation, and flow cytometry assays. The effect of MTMR3 knockdown on cell apoptosis was evaluated by flow cytometry with Annexin V/7-AAD double staining. The activation of apoptotic markers, Bad and PARP, was detected using Intracellular Signaling Array. Knockdown of MTMR3 resulted in a significant reduction in cell proliferation in both HCT116 and SW1116 cells. Moreover, knockdown of MTMR3 led to S phase cell cycle arrest. Furthermore, knockdown of MTMR3 induced cell apoptosis via phosphorylation of Bad and cleavage of PARP. These results indicate that MTMR3 may play an important role in the progression of CRC and suggest that siRNA mediated silencing of MTMR3 could be an effective tool in CRC treatment.

## 1. Introduction

As estimated, based on 2006–2010 data, there were 45 per 100,000 men and women diagnosed with colorectal cancer (CRC) and 16.4 per 100,000 people died of it every year according to the last update from the Surveillance Epidemiology and End Results (SEER) data from the National Cancer institute (NCI), making colorectal cancer one of the leading causes of morbidity and mortality from cancer in the word [1]. In spite of current efforts in understanding transition from healthy colonic epithelia to CRC, the overall prognosis is poor (20% of patients are diagnosed once their tumor has metastasized) [2] and the molecular events that lead to the development of this disease are still little known [3].

MTMR3 (myotubularin-related phosphatase 3) is a phosphoinositide (PI) phosphatase that belongs to the myotubularin (MTM) family, which are PI 3-phosphatases with specificity for phosphatidylinositol(3)-phosphate (PtdIns3P) and phosphatidylinositol(3,5)-biphosphate (PtdIns(3,5)P2) [4, 5]. It contains a PH-GRAM (PHG) domain at its N-terminal, which is necessary for MTMR3 binding to PI lipids. MTMR3 can hydrolyze PtdIns3P and PtdIns(3,5)P2 in vitro [4–6].

MTMR3 is a ubiquitously expressed myotubularin, which shows both cytosolic and reticular localisation upon overexpression, but its specific role is not very clear [5, 7]. Evidence shows that MTMR3 modulates the local PtdIns3P levels and negatively regulates autophagy. Knockdown of MTMR3 increased autophagosome formation, and overexpression of wild-type MTMR3 led to significantly smaller nascent autophagosomes and a net reduction in autophagic activity [7]. Yoo et al. reported that MTMR3 could negatively regulate the growth of lung cancer cells [8]. They found MTMR3 increased the cyclin-dependent kinase inhibitor, p27, and arrested cell-cycle at G1. Last year, a new role of MTMR3 was revealed in oral cancer. Kuo et al. found that MiR-99a exerts antimetastasis through inhibiting MTMR3 expression, making MTMR3 a therapeutic target for oral cancer treatment [9]. However, the functional role of MTMR3 in CRC is still unknown.

In this study, we investigated the role of MTMR3 in CRC cell growth using lentivirus-mediated small interfering RNA (siRNA) and demonstrated that MTMR3 silencing led to decreased cell proliferation, impaired colony formation, arrested cell cycle, and increased apoptosis.

## 2. Materials and Methods

### 2.1. Cell Culture

Human colon cancer cell lines HCT116 and SW1116 and human embryonic kidney cell line 293T were obtained from the Cell Bank of Chinese Academy of Science (Shanghai, China). HCT116 and SW1116 cells were cultured in McCoy's 5A medium (Sigma, USA) supplemented with 10% fetal bovine serum (FBS). 293T were cultured in DMEM (Hyclone, USA) with 10% FBS. Cells were incubated at 37°C in a humidified atmosphere with 5% CO_2_.

### 2.2. Construction of MTMR3 shRNA Lentiviral Vector

The short hairpin RNA (shRNA) sequence (5′-CCAGTCGAGTATGCAAGTCTTGGTACCAAGACTTGCATACTCGACTGG-3′) was designed based on human MTMR3 gene (NM_021090.3) and cloned into the pFH-L vector (Shanghai Hollybio, China). The sequence of nonsilencing control siRNA was 5′-TTCTCCGAACGTGTCACGT-3′. The lentiviral-based shRNA expressing vectors were confirmed by DNA sequencing.

### 2.3. Lentivirus Packing and Infection

Lentiviruses were generated by transfection of 293T cells at 80% confluence with modified pFH-L vector and packing plasmids pVSVG-I and pCMVΔR8.92 (Shanghai Hollybio, China) using Lipofectamine 2000, according to the manufacturer's instructions. At 48 h after transfection, supernatant was collected and lentiviral particles were harvested by ultracentrifugation (4000 g) at 4°C for 10 min, followed by filtration through 45 *μ*m filter.

For lentivirus infection, HCT116 and SW1116 cells (5 × 10^4^ cells/well) were seeded into 6-well plates and transduced with MTMR3 shRNA (Lv-shMTMR3) or control shRNA (Lv-shCon) expressing lentivirus at a multiplicity of infection (MOI) of 15 and 50, respectively. Infection efficiency was determined by measuring the number of red fluorescence protein- (RFP-) expressing cells under fluorescence microscope 72 h after infection.

### 2.4. RNA Isolation, Reverse Transcription, and Quantitative PCR (RT-qPCR)

Total RNA was extracted by Trizol Reagent (Invitrogen, USA) and reverse transcription was performed using Moloney murine leukemia virus (M-MLV) reverse transcriptase (Promega, number M1705), according to the manufacturer's instructions.

RT-qPCR was performed on Bio-Rad Connect Real-Time PCR platform with SYBR Green reagents (Bio-Rad, number 170-8882). The RT-qPCR analysis was performed in a total volume of 20 *μ*L with the following amplification steps: an initial denaturation step at 95°C for 1 min, followed by 40 cycles of denaturation at 95°C for 5 sec, and annealing extension at 60°C for 20 sec. The RT-qPCR gene expression was normalized to human *β*-actin. The primers used were as follows: 5′-AGCAGAGTGGGCTCAGTGTT-3′ (sense) and 5′-ACTGTCCACGTTTGGTCCTC-3′ (antisense) for MTMR3; 5′-GTGGACATCCGCAAAGAC-3′ (sense) and 5′-AAAGGGTGTAACGCAACTA-3′ (antisense) for *β*-actin.

### 2.5. Western Blotting

After infection for 5 days, HCT116 and SW1116 cells were washed with cold PBS and lysed in 2X SDS sample buffer (100 mM Tris-HCl (pH 6.8), 10 mM EDTA, 4% SDS, and 10% glycine) for 1 h at 4°C, respectively. After centrifuging (12,000 ×g for 15 min), the protein content was measured by the enhanced BCA protein assay kit (Beyotime). Equal amounts (30 *μ*g) of protein in each lane were separated by 10% SDS-PAGE and transferred to a PVDF membrane (Millipore, Billerica, MA, USA). Membranes were blocked and then incubated with primary antibodies: rabbit anti-MTMR3 (1 : 1000, Cell Signaling, number 12443) and mouse anti-GAPDH (1 : 60000, Santa Cruz, number Sc-32233) overnight at 4°C. After washing with TBST, the blots were incubated with HRP-labeled anti-rabbit (1 : 5000, Santa Cruz, number Sc-2054) or anti-mouse (1 : 5000, Santa Cruz, number Sc-2005) secondary antibody for 2 h at room temperature and then visualized by super ECL detection reagent (Applygen, Beijing, China).

### 2.6. MTT Assay

To detect the antiproliferative effect of MTMR3 shRNA, an MTT assay was performed in both HCT116 and SW1116 cells. After 96 h of infection, HCT116 and SW1116 cells were seeded in 96-well plates at a density of 2.5 × 10^3^ cells/well and incubated for 1, 2, 3, 4, or 5 days. At each time point, 20 mL of 5 mg/mL MTT solution (Sigma) was added to each well. After 4 h of incubation, acidic isopropanol (10% SDS, 5% isopropanol, and 0.01 mol/L HCl) was added to stop the reaction and measured with an ELISA reader (Bio-Rad, Hercules, CA, USA) at a wavelength of 595 nm.

### 2.7. Colony Formation Assay

To detect the long-term antiproliferative effect of MTMR3 shRNA, a colony formation assay was performed in HCT116 cells. Lentivirus infected HCT116 cells were seeded in triplicate at an initial concentration of 400 cells/well in 6-well plates. After 9 days of culture, the cells were washed and fixed by paraformaldehyde. Fixed cells were washed twice with PBS solution, treated with crystals purple for 10 min, washed 3 times with ddH_2_O, and then photographed with a digital camera. The number of colonies (>50 cells/colony) was counted.

### 2.8. Flow Cytometry Analysis of Cell Cycle

The cell cycle progression was analyzed by flow cytometry using propidium iodide (PI) staining. HCT116 cells were cultured in 6-well plates and inoculated with recombinant lentiviruses at an MOI of 8. After 5 days of infection, cells were inoculated into 6 cm dishes at a density of 1 × 10^5^ cells/dish. After 120 h of incubation, HCT116 cells were digested with trypsin and centrifuged, washed with cold PBS twice, and fixed in precold 70% ethanol at −20°C overnight. The fixed cells were resuspended in PI/RNase/PBS buffer for incubation in dark (37°C, 30 min). Then, samples were detected by flow cytometer (Cell Lab Quanta, Beckman Coulter) and the data were analyzed using Multi-Cycle AV software (Phoenix Flow Systems, San Diego, CA).

### 2.9. Flow Cytometry Analysis of Apoptosis

The cell apoptosis was analyzed by flow cytometry using Annexin V/7-AAD double staining. In brief, lentivirus infected HCT116 cells were reseeded in 6 cm dishes at a density of 1 × 10^5^ cells/dish. Then cells were collected and subjected to Annexin V-APC/7-AAD double staining according to the manufacture's instruction (KeyGEN Biotech, number KGA1026).

### 2.10. Intracellular Signaling Array

Intracellular Signaling Array was performed using a PathScan Intracellular Signaling Array Kit (Cell Signaling Technology, number 7323), which allows for the simultaneous detection of 18 important and well-characterized signaling molecules when phosphorylated or cleaved. HCT116 cells were collected and washed once with ice-cold PBS following addition of ice-cold cell lysis buffer. The lysate was then collected into a clean microtube and intended for the assay. The Intracellular Signaling Array was performed according to the protocol provided by CST.

### 2.11. Statistical Analysis

All results represent the mean ± standard deviation from three independent experiments. The Student's* t*-test was used to evaluate the differences between groups using SPSS 13.0 software. Significant significance was set at *P* < 0.05.

## 3. Results

### 3.1. Effective Knockdown of MTMR3 by shRNA in Colon Cancer Cells

**Figure 1:**
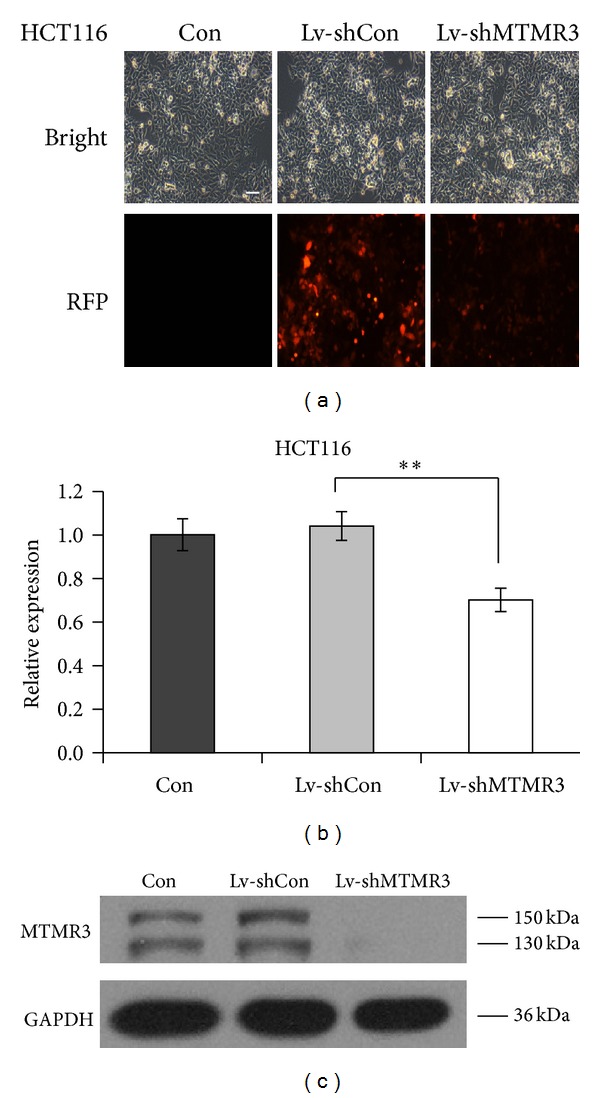
Verification of MTMR3 silencing in HCT116 cells after Lv-shMTMR3 infection. (a) Transduction efficiency was assessed by counting RFP-expressing cells with fluorescence microscopy (scale bar: 50 *μ*m). (b) The mRNA levels of MTMR3 in HCT116 cells with three treatments (Lv-shMTMR3, Lv-shCon, and Con) measured by RT-qPCR. The experiment was performed in triplicate and repeated three times. (c) The protein levels of MTMR3 in HCT116 cells with three treatments (Lv-shMTMR3, Lv-shCon, and Con) measured by western blot. The experiment was repeated three times (**P < 0.01).

HCT116 and SW1116 cells were successfully infected with the Lv-shMTMR3 or Lv-shCon, as confirmed by evaluating the expression of RFP. As shown in Figure 1(a) and Supplementary Figure S1a (see Supplementary Material available online at http://dx.doi.org/10.1155/2014/703804), the delivery efficiency of recombined lentiviruses was more than 80%. Furthermore, the gene silencing of MTMR3 was evaluated by RT-qPCR and western blotting analysis. As shown in Figures 1(b) and 1(c) and Supplementary Figure S1b, infection of lentivirus containing MTMR3 shRNA significantly reduced both the mRNA and the protein levels of MTMR3 in HCT116 and SW1116 cells. In contrast, the nonsilencing shRNA had no effect on MTMR3 expression, confirming that MTMR3 expression was specifically decreased by MTMR3 shRNA.

### 3.2. MTMR3 Knockdown Leads to a Decline in Cell Proliferation and Colony Formation

**Figure 2:**
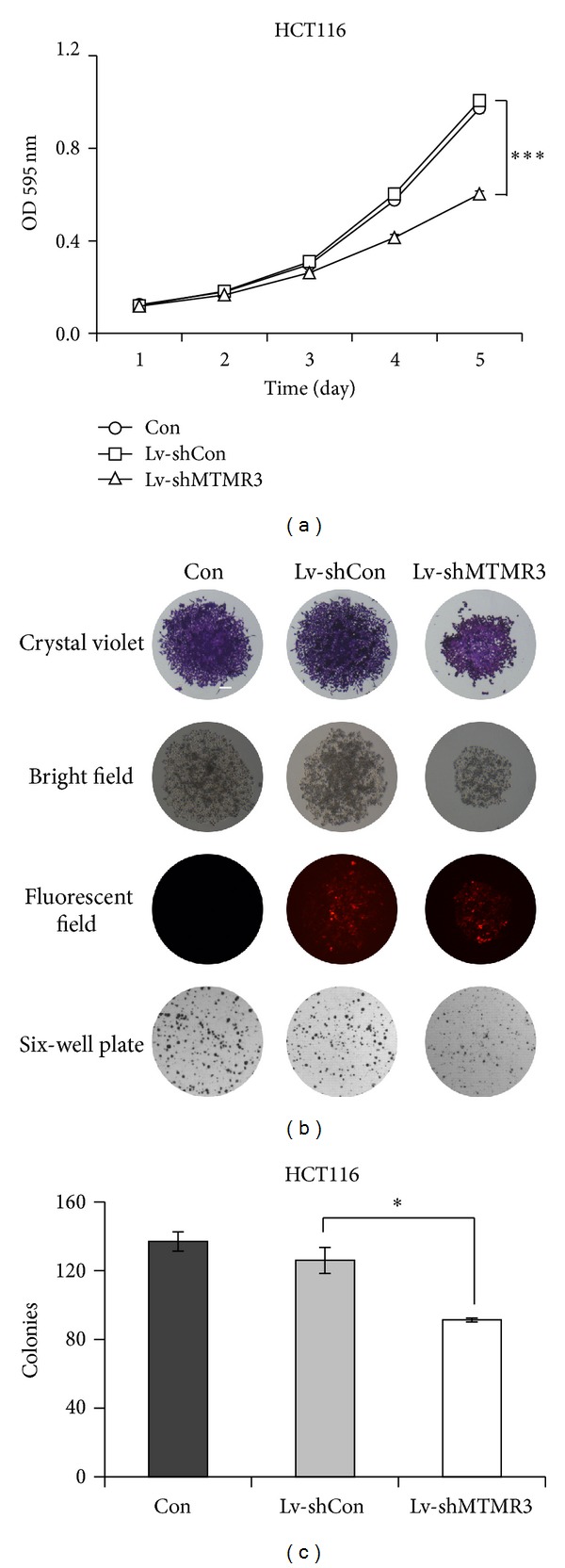
Lentivirus-mediated knockdown of MTMR3 suppresses the proliferation and the colony formation ability of HCT116 cells. (a) Growth curve of HCT116 cells with three treatments (Lv-shMTMR3, Lv-shCon, and Con) measured by the MTT assay. The experiment was performed in triplicate and repeated three times. (b) Representative images of colonies formed in HCT116 cells with three treatments (Lv-shMTMR3, Lv-shCon, and Con) examined by the colony formation assay (scale bar: 125 *μ*m). The experiment was repeated three times. (c) Statistic results of the colony formation ability as shown (*P < 0.05, ***P < 0.001).

After confirming the knockdown efficiency of the shRNA targeting MTMR3, we determined the effect of a reduced MTMR3 level on cell proliferation using MTT assay. As shown in Figure 2(a), the proliferation rate of HCT116 cells was significantly decreased in a time-dependent manner after Lv-shMTMR3 infection. However, nonsilencing cells had no obvious difference concerning cell proliferation against control cells. The similar result was clearly observed in SW1116 cells (Figure S1c). These results demonstrated that the MTMR3 had a positive effect on the proliferation of colon cancer cells.

Meanwhile, we evaluated the colony formation capacity in HCT116 cells with three different treatments (Lv-shMTMR3, Lv-shCon, and Con), which represents a loss of contact inhibition or the ability to maintain cell growth and movement despite contact with surrounding cells [10]. Both the size of single colony and the number of colonies formed in HCT116 cells were remarkably decreased after Lv-shMTMR3 infection (Figure 2(b)). As shown in Figure 2(c), the colonies numbers in the Lv-shMTMR3 group (91.3 ± 1.2) were significantly lower than those in Lv-shCon (126 ± 7.5) and Con (137.0 ± 5.6) groups. Collectively, these results strongly support that MTMR3 is essential for the growth of HCT116 colon cancer cells.

### 3.3. MTMR3 Knockdown Induces S Phase Cell Cycle Arrest

**Figure 3:**
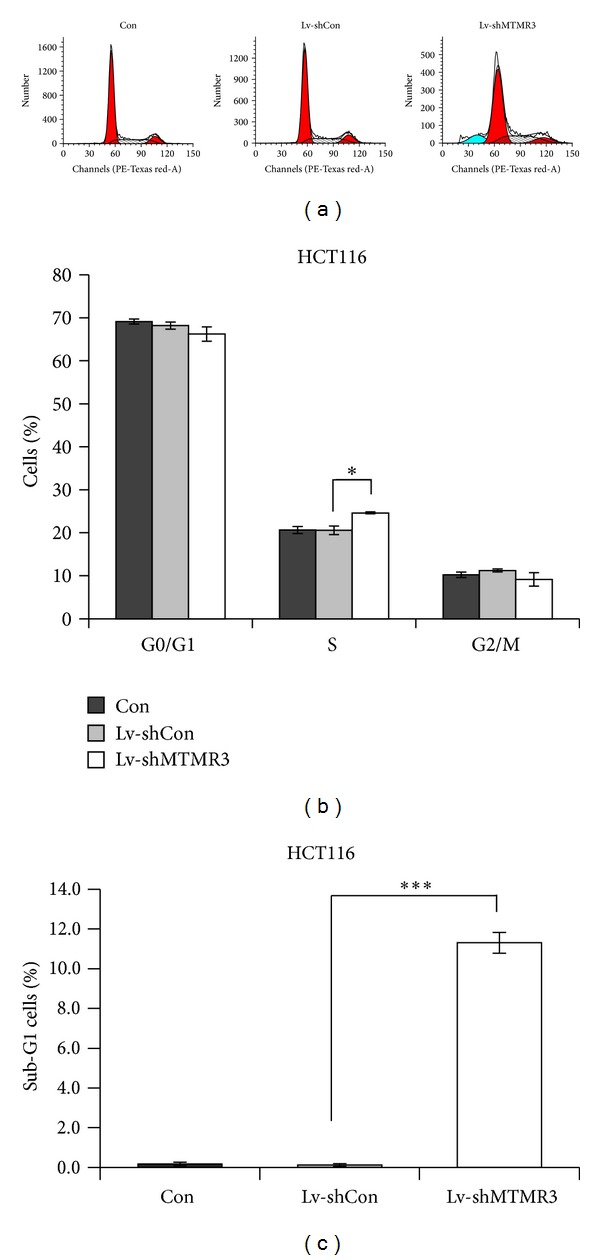
Lentivirus-mediated knockdown of MTMR3 blocks the cell cycle progression of HCT116 cells. (a) Cell cycle distribution of HCT116 cells with three treatments (Lv-shMTMR3, Lv-shCon, and Con) analyzed by flow cytometry using PI staining. The experiment was performed in triplicate and repeated three times. (b) The respective proportion of HCT116 cells in the G0/G1 phase, S-phase, and G2/M phase. (c) The respective proportion of HCT116 cells in the sub-G1 phase. (*P < 0.05, ***P < 0.001).

To explore the potential mechanism of cell growth inhibition, flow cytometry was performed to determine the cell cycle distribution of HCT116 cells with three different treatments (Lv-shMTMR3, Lv-shCon, and Con) (Figure 3(a)). The cell proportion of S phase increased from 20.58% in the Lv-shCon group to 24.62% in the Lv-shMTMR3 group, which meant that a small part of HCT116 cells was arrested in the S phase (*P* < 0.05, Figure 3(b)). In addition, as shown in Figure 3(c), MTMR3 knockdown in HCT116 cells also led to a significant accumulation in the sub-G1 phase (*P* < 0.001), which represents apoptotic cells [11]. These results suggest that the reduction of MTMR3 expression in HCT116 cells by shRNA delays cell cycle progression and induces apoptosis, thereby resulting in a decline in cell proliferation.

### 3.4. MTMR3 Knockdown Induces Cell Apoptosis via Phosphorylation of Bad and Cleavage of PARP

**Figure 4:**
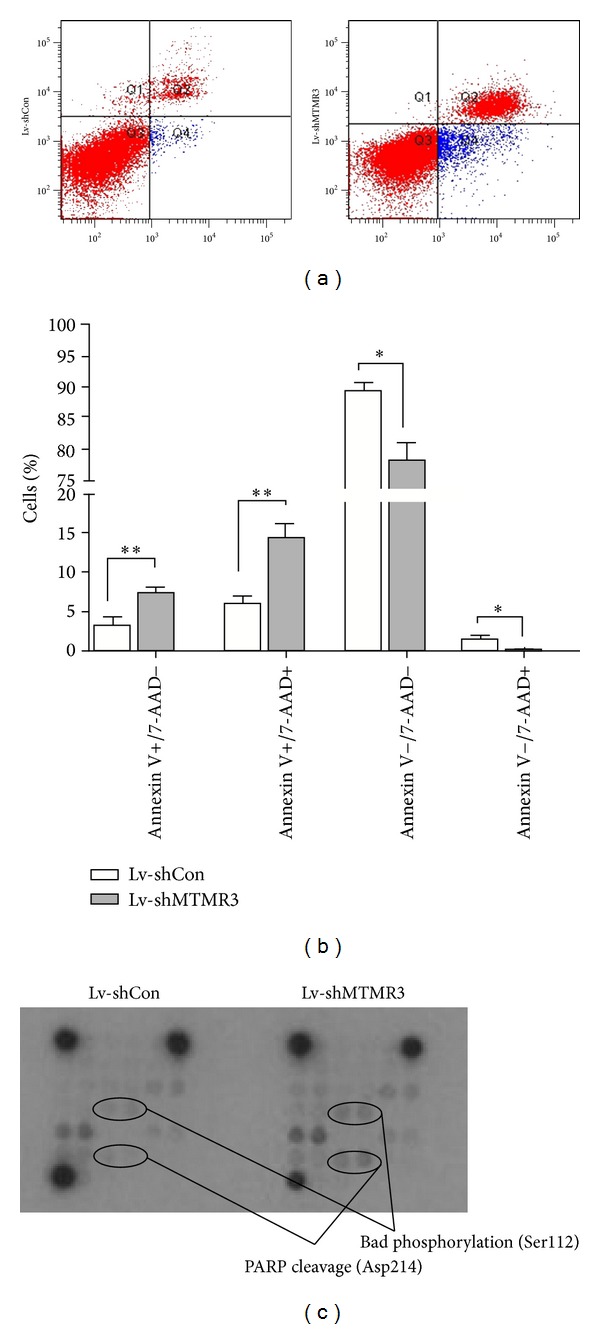
Knockdown of MTMR3 induces cell apoptosis via activation of Bad and PARP signaling pathways. (a) Cell apoptosis of HCT116 cells with two treatments (Lv-shMTMR3 and Lv-shCon) analyzed by flow cytometry using Annexin V/7-AAD double staining. The experiment was performed in triplicate and repeated three times. (b) The proportions of HCT116 cells corresponding to viable (Annexin V−/7-AAD−), necrotic (Annexin V−/7-AAD+), early (Annexin V+/7-AAD−), and late (Annexin V+/7-AAD+) apoptotic cells. (c) Activation of Bad and PARP signaling pathways measured by Intracellular Signaling Array. (*P < 0.05, **P < 0.01).

To further investigate the mechanism underlying the cell growth inhibition, we applied Annexin V/7-AAD double staining in HCT116 cells following lentivirus infection (Figure 4(a)). Annexin V versus 7-AAD plots from the gated cells showed the populations corresponding to viable (Annexin V−/7-AAD−), necrotic (Annexin V−/7-AAD+), early (Annexin V+/7-AAD−), and late (Annexin V+/7-AAD+) apoptotic cells. Apoptotic cells (early apoptosis and late apoptosis) were significantly augmented in the Lv-shMTMR3 group, as compared to the Lv-shCon (Figure 4(b)). The results revealed that knockdown of MTMR3 induced a strong proapoptotic effect in colon cancer cells. Furthermore, to explore the underlying signaling pathways mediated by MTMR3 in colon cancer cells, we examined 18 important and well-characterized signaling molecules using PathScan Intracellular Signaling Array kit. Using this antibody-based approach, we screened for the phosphorylation or cleavage of several cellular proteins and signaling nodes in downstream signaling pathways after the treatment of HCT116 cells with Lv-shMTMR3 against Lv-shCon. As shown in Figure 4(c), the expression levels of phosphorylated Bad and cleaved PARP (poly ADP-ribose polymerase) were significantly upregulated compared with the Lv-shCon group. These results revealed that knockdown of MTMR3 induced cell apoptosis via phosphorylation of Bad and cleavage of PARP. Thus, we could infer that MTMR3 knockdown suppressed colon cancer cell growth through inducing cell cycle arrest and apoptosis.

## 4. Discussion

Due to changes in lifestyle, particularly changes in dietary habits, colorectal cancer increased in recent years in Asia in spite of the higher treatment on controlling the cancer progression [12, 13]. As one of leading causes of cancer mortality, CRC has nearly one million new cases diagnosed worldwide and half a million deaths each year [14]. Therefore, novel methods of cancer treatments are approached recently. Among them cancer therapy at the gene level through understanding of the molecular functions of cancer cell survival has gained more attention [14, 15]. This study was focused on identification of an oncogenic target in CRC and investigation of the effects of silencing the respective gene on CRC cell proliferation.

In our study, we presented data supporting a novel role for the myotubularin-related phosphatase MTMR3 in CRC. The effects of MTMR3 on proliferation of cultured cells showed diverse effects distinct from previously published studies with different sources of cells. For example, Yoo et al. found that MTMR3 negatively regulates the growth of lung cancer cells by increasing the cyclin-dependent kinase inhibitor, p27, and arresting cell cycle at G1 [8]. And a new role of MTMR3 was revealed in oral cancer last year as a downstream regulator of MiR-99a in the antimetastasis process [9]. These results indicated the potential effect of MTMR3 in the prevention and treatment of cancer. For this reason, it may be meaningful to better understand the regulatory activity of MTMR3 on the proliferation of CRC and comprehensively evaluate the effect in all its bearings of MTMR3. Unrestricted division and proliferation are important features of tumor cells. Detection of an inhibitory effect on tumor cell proliferation is a basic index for study of a gene function. Here we reported that the cell growth ability was markedly declined when MTMR3 expression was inhibited in HCT116 and SW1116 cells by specific MTMR3 shRNA, suggesting MTMR3 as a potential target for CRC therapy.

Abnormality of cell cycle regulation is one of intrinsic factors for tumor occurrence [16]. After MTMR3 silencing, cell cycle was arrested in the S phase. Meanwhile, a distinct increase of cell population in the sub-G1 phase indicated the induction of apoptosis. Moreover, flow cytometry analysis showed that knockdown of MTMR3 induced cell apoptosis, which contributed to cell growth inhibition. Previous studies showed that activation of Bad or PARP inhibits cell proliferation and promotes apoptosis while inhibition of them increases cell growth rate [17, 18]. In this study, the expression levels of phosphorylated Bad and cleaved PARP were found to be increased in HCT116 cells after MTMR3 silencing, suggesting activation of the intracellular apoptotic pathway [19].

Apoptosis is a form of programmed cell death (PCD) [20]. The process of apoptosis and the clearance of apoptotic cells are one of the most important factors for maintaining body health. We demonstrated that knockdown of MTMR3 induced cell apoptosis via phosphorylation of Bad and cleavage of PARP. However, autophagy, classified as type II PCD, also can initiate characteristic cell death under some circumstances [21, 22]. And it has been demonstrated that MTMR3 negatively regulates autophagy by knockdown of MTMR3 increasing autophagosome formation [7]. Autophagy controls a wide range of physiological processes such as starvation, cell differentiation, cell survival, and death [21–24] by the turnover of long-lived proteins, the disposal of damaged organelles and misfolded proteins, and the turnover of cellular building blocks following nutrient deprivation [25]. Though autophagy seems a double-edged sword that plays prosurvival role whilst also acting as a cell death mechanism [26], several lines of evidence suggested that autophagy and apoptosis can coexist or occur sequentially to induce cell death [27–30]. But the promising relationship of MTMR3-shRNA induced growth reduction and autophagy needs further study.

## 5. Conclusions

In this study, we identify MTMR3 as a critical gene in the colon cancer cell survival and growth. siRNA mediated silencing of MTMR3 has an inhibitory effect on the proliferation of colon cancer cells by inducing cell cycle arrest and apoptosis. Accordingly, we suggested that the intervene strategy targeting MTMR3 in CRC may be of clinical value.

## Supplementary Material

Response: Furthermore, we verified our results in another CRC cell line SW1116. Lv-shMTMR3 also efficiently transduced into SW1116 cells, as revealed by RFP fluorescence in Supplementary Figure S1a. The expression of MTMR3 was obviously decreased in SW1116 cells after Lv-shMTMR3 infection (Supplementary Figure S1b). The proliferation rate of SW1116 cells was also markedly decreased by MTMR3 knockdown (Supplementary Figure S1c). Taken together, these results indicated that knockdown of MTMR3 could significantly inhibit CRC cell proliferation.
