# Supplementary figures and images for: Myotubularin-Related Phosphatase 3 Promotes Growth of Colorectal Cancer Cells
Source: ScientificWorldJournal. 2014 Aug 19;2014:703804. doi: 10.1155/2014/703804 (PMC4152950; doi:10.1155/2014/703804)

**A**

**Con**

**Lv-shCon**

**Lv-shMTMR3**


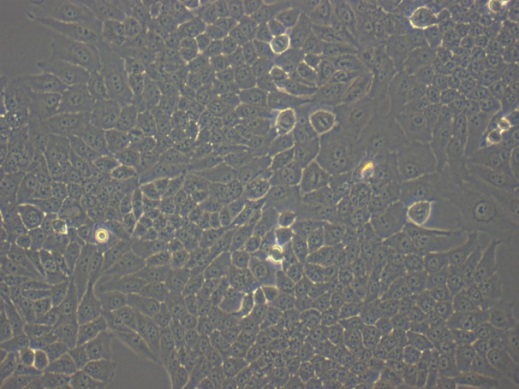

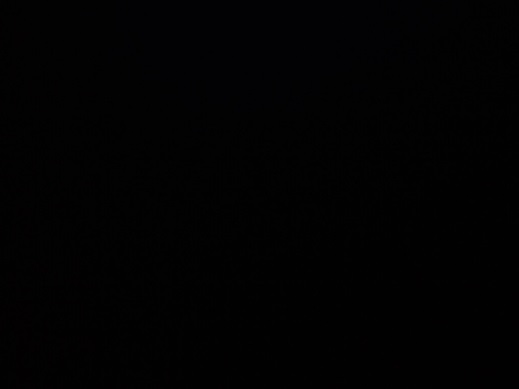

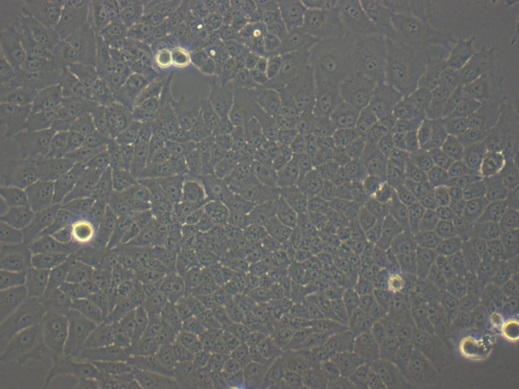

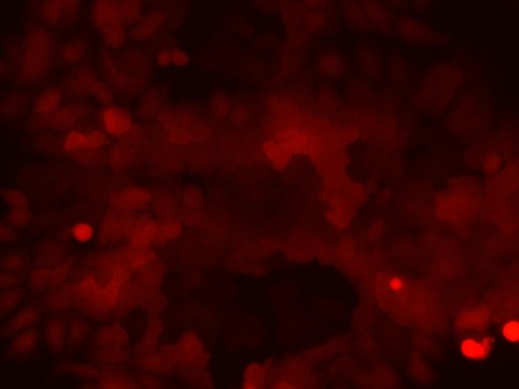

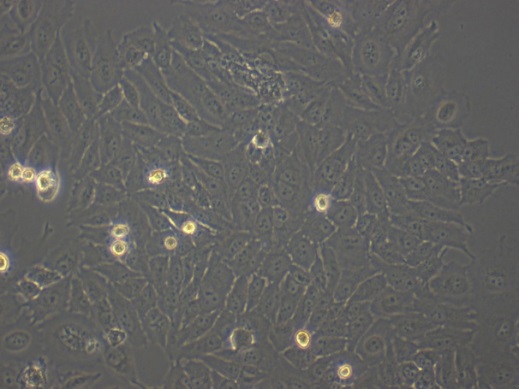

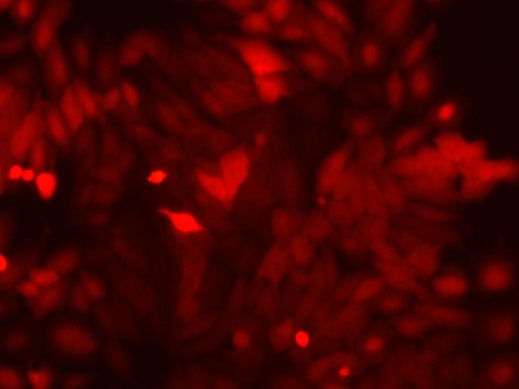


**Bright**

**RFP**

**B**

**Lv-shCon**

**Lv-shMTMR3**

**Con**


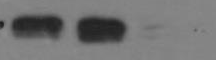


**MTMR3**

**130 kDa**

**GAPDH**


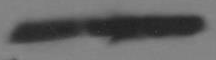


**36 kDa**

**C**


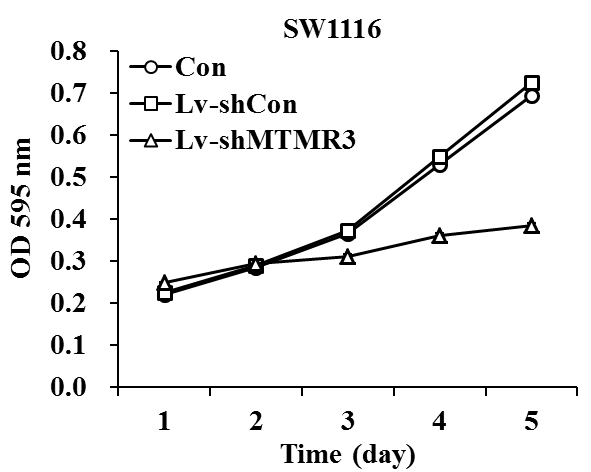

Supplement: Supplementary file 1 — Response: Furthermore, we verified our results in another CRC cell line SW1116. Lv-shMTMR3 also efficiently transduced into SW1116 cells, as revealed by RFP fluorescence in Supplementary Figure S1a. The expression of MTMR3 was obviously decreased in SW1116 cells after Lv-shMTMR3 infection (Supplementary Figure S1b). The proliferation rate of SW1116 cells was also markedly decreased by MTMR3 knockdown (Supplementary Figure S1c). Taken together, these results indicated that knockdown of MTMR3 could significantly inhibit CRC cell proliferation. [file 703804.f1.doc]
